# Electrographic seizures and brain hyperoxia may be key etiological factors for postconcussive deficits

**DOI:** 10.1152/jn.00533.2021

**Published:** 2022-08-16

**Authors:** Haris Malik, Marshal D. Wolff, G. Campbell Teskey, Richelle Mychasiuk

**Affiliations:** ^1^Hotchkiss Brain Institute, University of Calgary, Calgary, Alberta, Canada; ^2^Department of Cell Biology and Anatomy, University of Calgary, Calgary, Alberta, Canada; ^3^Department of Psychology, University of Calgary, Calgary, Alberta, Canada; ^4^Department of Neuroscience, Monash University, Melbourne, Victoria, Australia

**Keywords:** Bay K8644, hippocampus, mild traumatic brain injury, postictal, vasculature

## Abstract

Repetitive mild traumatic brain injuries (RmTBIs) are increasingly recognized to have long-term neurological sequelae in a significant proportion of patients. Individuals that have had RmTBIs exhibit a variety of sensory, cognitive, or behavioral consequences that can negatively impact quality of life. Brain tissue oxygen levels (${\text{P}}_{{\text{O}}_{2}}$) are normally maintained through exquisite regulation of blood supply to stay within the normoxic zone (18–30 mmHg in the rat hippocampus). However, during neurological events in which brain tissue oxygen levels leave the normoxic zone, neuronal dysfunction and behavioral deficits have been observed, and are frequently related to poorer prognoses. The oxygenation response in the brain after RmTBIs/repeated concussions has been poorly characterized, with most preliminary research limited to the neocortex. Furthermore, the mechanisms by which RmTBIs impact changes to brain oxygenation and vice versa remain to be determined. In the current study, we demonstrate that upon receiving RmTBIs, rats exhibit posttraumatic, electrographic seizures in the hippocampus, without behavioral (clinical) seizures, that are accompanied by a long-lasting period of hyperoxygenation. These electrographic seizures and the ensuing hyperoxic episodes are associated with deficits in working memory and motor coordination that were reversible through attenuation of the posttraumatic and postictal (postseizure) hyperoxia, via administration of a vasoconstricting agent, the calcium channel agonist Bay K8644. We propose that the posttraumatic period characterized by brain oxygenation levels well above the normoxic zone, may be the basis for some of the common symptoms associated with RmTBIs.

**NEW & NOTEWORTHY** We monitor oxygenation and electrographic activity in the hippocampus, immediately before and after mild traumatic brain injury. We demonstrate that as the number of injuries increases from 1 to 3, the proportion of rats that exhibit electrographic seizures and hyperoxia increases. Moreover, the presence of electrographic seizures and hyperoxia are associated with postinjury behavioral impairments, and if the hyperoxia is blocked with Bay K8644, the behavioral deficits are eliminated.

## Graphical abstract

**Figure:**
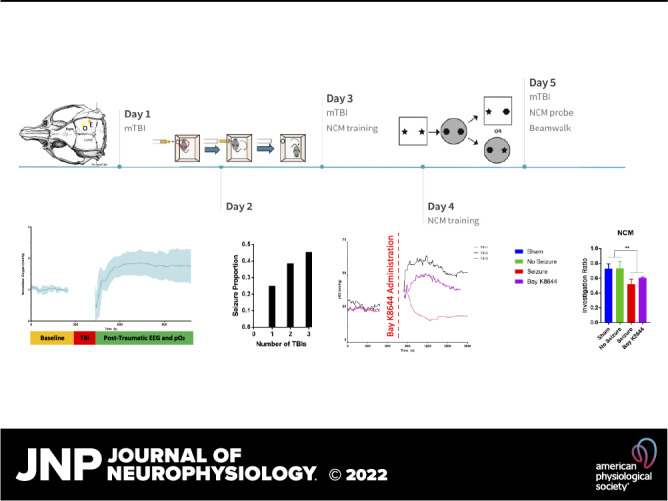

## INTRODUCTION

Traumatic brain injuries (TBIs) are a set of injuries that involve an insult to the brain through the application of an external mechanical force to the head or body that can give rise to neuropathologic damage and dysfunction (1). Of all severities of TBI, an estimated 75%–85% are categorized as mild TBI (mTBI), which includes concussions (2). Although the secondary injury cascades associated with mTBI are usually self-limiting and normally resolve themselves over the course of several weeks, 15%–30% of individuals develop prolonged neurocognitive and behavioral changes referred to as postconcussive syndrome (3, 4). Repetitive mTBIs (RmTBIs) in particular, are increasingly recognized to increase the risk for postconcussive syndrome and induce long-term neurological sequelae in a significant proportion of patients (5, 6). Although animal models of mTBI have elucidated a metabolic cascade of events in the acute phase following injury, it remains unclear how these metabolic events cause the behavioral deficits identified (7).

Although there have been several studies assessing brain electrical activity in the subacute to late period after mTBI, very few studies have investigated brain activity immediately after such an event (8). Although there are no published studies of human EEG during the actual injury event, Dow et al. (8) recorded EEG as soon as 10–15 min after a closed head injury and found that some individuals had diminution of electrical activity that resolved quickly (e.g., within an hour), with the signal diminution being especially prominent in those recordings made soonest after injury. In addition, a study by Korinthenberg et al. (9) found that EEG patterns in the acute period following a single mTBI failed to predict postconcussive syndrome in children. However, to our knowledge, no studies have examined the relationship between epileptiform EEG patterns and neurological examination scores in the immediate and acute phases following RmTBIs. The scarcity of observation of brain activity and simultaneous assessment of symptomology in the acute phase of injury presents a gap in the literature that may shed light on the relationship between electrophysiologic abnormalities and behavioral dysfunction after RmTBI.

It has previously been hypothesized that the loss of consciousness and other functional impairments that follow mTBIs are due to a brief episode of vascular dysfunction or dysregulation (10). However, the mechanisms triggering these vascular events are currently uncertain. Importantly, behavioral seizures have been noted in response to moderate and severe TBI (11), and are often associated with region-specific changes in cerebral blood flow (12–15). Given that more recent seizure studies have reported local brain oxygen levels well below or above the normoxic zone, which are accompanied by behavioral consequences (16–18), it is possible that posttraumatic electrographic seizure activity could be driving changes in cerebral vascular supply in mTBI (19). Consequently, we examined EEG characteristics and evaluated corresponding changes in brain oxygenation in the hippocampus, a structure that is particularly vulnerable to injury, has a low seizure threshold, and often manifests electrographic seizure activity following injury without behavioral/clinical seizures (20). In addition, we examined behavioral function in the acute (immediate) phase after the injury.

Male Wistar rats were chronically implanted with a bipolar electrode and an oxygen sensing probe (optode) in the ventral and dorsal hippocampus, respectfully, 1 wk before receiving 3 mTBIs over 5 days using our lateral impact device (21). We acknowledge that the presence of an indwelling electrode and optode may also have contributed to the outcomes and altered the biomechanics of brain movement, but this approach was the only way to properly record hippocampal EEG and oxygen levels immediately before and after mTBI in awake behaving rats. Immediately following induction of the third and final injury, behavioral outcomes were measured in a subset of animals, 15 min postinjury, using tasks designed to examine motor and cognitive symptoms consistent with clinical signs of RmTBI (22). These experiments were used to evaluate the relationship between posttraumatic electrographic activity, local oxygen levels, and behavioral function. To prevent vasodilation-induced increases in oxygen levels in rats post-RmTBI, BAY K8644, an L-type calcium channel agonist, was used to induce vasoconstriction in an effort to restrict posttraumatic oxygen levels to be within the normoxic zone and restore typical behavior (23).

## METHODS

### Rats

**Figure 1.**
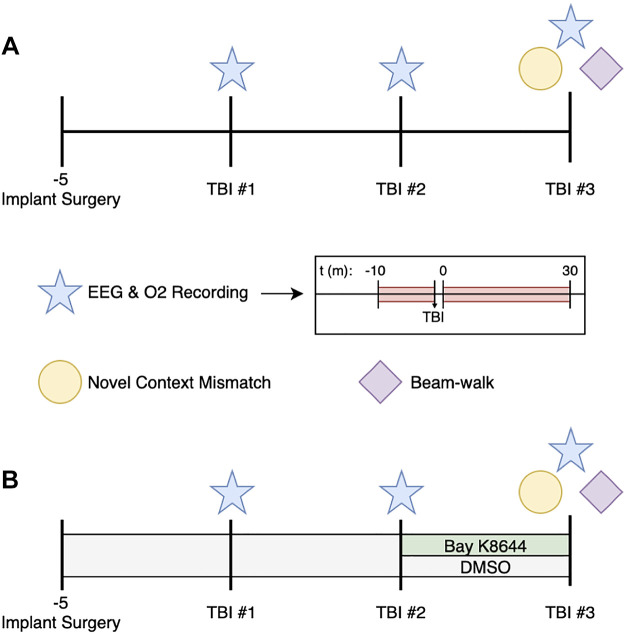
Schematic timeline for experimental procedures. *A*: the procedures and group sizes for the first set of experiments designed to characterize the phenomena. *B*: the procedures and group sizes for the experiments used in the Bay K8644 manipulation study. TBI, traumatic brain injury.

Forty-six young adult male Wistar rats weighing between 250 and 300 g at the start of experimentation were used in this study (Charles River, Canada). Rats were housed individually in standard shoe-box cages and were maintained on a 12:12-h light/dark cycle, lights on at 0700. Food and water were available ad libitum. All experimental procedures occurred during the light phase, beginning at 0900. The experiments and procedures were approved by the University of Calgary Conjoint Facilities Research Ethics Board and conducted in accordance with the Canadian Council of Animal Care and the 2020 ARRIVE guidelines (Animal Research: Reporting of In Vivo Experiments). See Fig. 1 for an illustrative timeline of all experimental procedures.

### Implant Procedure

Electrodes were constructed from Teflon-coated, stainless steel wire, 178 µm in diameter (A-M Systems, Sequim, WA). Wire ends were stripped of Teflon and crimped to gold-plated male amphenol pins. The electrodes and oxygen-sensing probes were implanted 7 days before administration of the mTBIs, so electrographic and oxygenation data could be obtained immediately before and following injury induction. Rats were anesthetized with 5% isoflurane and then maintained with between 1% and 2% isoflurane for the remainder of the surgery. Lidocaine (2%) was administered subcutaneously at the incision site to alleviate suffering. Based on bregma, one bipolar electrode was chronically implanted under stereotaxic control in the ventral hippocampus (posterior 5.6 mm; right 5.2 mm; and 7 mm deep) with an oxygen-sensing optode (Oxford Optronix Ltd.) positioned in the dorsal hippocampus (posterior 3.5 mm; right 3.5 mm; 3.5 mm deep). As the impacts from the RmTBI were delivered to the left hemisphere, the optode and electrode were implanted into the right hemisphere, contralateral to the impact site, to reduce the risk of implant movement. The implants were adhered and anchored to the skull using dental cement and four stainless steel screws. One of the four screws served as a ground electrode.

### RmTBI Procedure

Repetitive injuries, designed to mimic the biomechanical forces involved in sports-related concussion, were administered using our lateral impact device, as described previously (21). Rats were subjected to three injury procedures spaced one day apart. On injury days, rats were connected to the EEG and oxygen sensing system in a Faraday cage and allowed 15 min to adjust to the cage before taking a 10-min baseline recording of both metrics. They were then anesthetized using inhalant isoflurane gas (5% isoflurane in 100% O_2_ delivered at 1 L/min) until they were no longer responsive to a toe pinch (∼30 s) before being placed in flexible plastic restraint bag. Next, they were placed in the prone position on a Teflon board with the left side of the head facing the lateral impactor device. The animal was permitted to wake from the anesthesia in the restraint bag to ensure that the anesthetic did not interfere with electrographic seizure recordings. A 50-g cylindrical weight was propelled toward the left side of the head at 12 m/s (∼122 Gs) using a pneumatic air compressed barrel. The weight made impact with a small aluminum plate placed against the rat’s head. The purpose of this plate was to prevent bone or skull damage, while still ensuring the production of rotational, acceleration, and deceleration forces. Immediately after delivery of the injury, rats were reconnected to the EEG and oxygen sensing system and recorded for a further 30 min. The RmTBI procedure did not result in any mortality in this study. Sham rats were also anesthetized using inhalant isoflurane gas (5% isoflurane in 100% O_2_ delivered at 1 L/min) until they were no longer responsive to a toe pinch (∼30 s) before being placed in flexible plastic restraint bag. Sham animals were placed in the prone position on the Teflon board of the lateral impactor device with the left side of the head facing the piston. When the animal awoke from anesthesia in the restraint bag, it was immediately removed from the bag and reconnected to the EEG and oxygen sensing system.

### Drug Delivery

A subset of the rats implanted with ventral hippocampal electrodes and dorsal hippocampal optodes were used in this study (*n* = 35). Bay K8644 was obtained from Sigma-Aldrich and dissolved in 100% DMSO. Bay K8644 dosage was selected based upon previous studies using Bay K8644 and similar calcium channel modulators (16, 24). Drug administration (1 mg/kg dissolved in DMSO 1 mL/kg) was performed intraperitoneally immediately before or after delivery of the third mTBI. The vehicle control group was given equal volumes of DMSO. As there were no differences in oxygenation or behavioral outcomes associated with drug delivery timing (immediately before or immediately after the third mTBI), they were combined into a single “Bay K8644 drug” group for analyses.

### Behavioral Testing

Behavioral tests were chosen to model two symptoms (motor and cognitive impairment) commonly associated with mTBI. Fifteen minutes following delivery of the final mTBI, rats completed the novel context mismatch task to assess short-term working memory performance, which was immediately followed by the beam-walk task to measure motor performance, namely balance and coordination. All testing and habituation occurred in the light phase (between 1300 and 1800) and researcher blinded to experimental conditions scored the behavioral tests.

#### Novel context mismatch.

The Novel Context Mismatch (NCM) has been used a measure of short-term working memory, a hippocampal dependent task (25). For the habituation days, rats were placed in two different contexts (*context A* and *B*) for 5 min each, one context immediately preceding the other. *Context A* was a hexagonal, black bin in a dark room containing two identical objects. *Context B* was a square, white bin in a brightly lit room containing a different pair of identical objects. On the probe day, 20 min after delivery of the third mTBI, rats underwent a probe trial; *context A* (5 min) → *context B* (5 min) → home-cage (5 min) → novel context (5 min). The novel context consisted of a modified *context A* or *B* (counterbalanced such that half the rats were exposed to modified A and half were exposed to modified B), containing one object from *context A* and one object from *context B*. The novel object for each modified context was the one originally located in the opposing context. Exploration of the novel context was video recorded, and the amount of time each rat spent investigating the novel object and the familiar object was determined. All the objects and context containers were cleaned with Virkon between each testing session.

#### Beam walk.

Following completion of the novel context mismatch task, rats were placed in their home cage for 5 min before undertaking the beam-walk task. This procedure was designed to assess the balance and motor function impairments that are often seen following concussion (26). Rats were placed at one end of a 165-cm long tapered beam with the wide end at the start and the narrower end placed in their home cage. The beam was suspended between two platforms ∼1 m off the ground and had 2 cm ledges that catch the hind legs if the rat slipped off the central portion of the beam. Each rat underwent 1 unscored pretraining trial. The following four trials were videotaped and scored. Hind leg foot slips were scored every time the rat used the safety ledge with the rear foot while moving across the beam. The beam was cleaned with Virkon between each rat.

Following completion of behavioral testing, rats were administered a lethal dose of pentobarbitone (160 mg/kg) and once no longer responsive to a strong toe pinch, they were rapidly decapitated.

### Data Acquisition and Analysis

The EEG signals were filtered, at half amplitude, below 0.5 Hz and above 300 Hz and then amplified 500 or 1,000 times (Grass Neurodata Acquisition System Model 12) and recorded with Windaq Recording Software (Dataq Instruments). EEG activity was analyzed using a fast Fourier transform (FFT) algorithm (SUDSA22 script, Spike2 software; CED). Power spectra were generated for every 60 s epoch of the EEG through the analysis of overlapping 1,024-sample segments that were windowed with a raised cosine (Hanning) and subjected to an FFT. Each 60 s epoch was then analyzed for average absolute power in five bandwidths: (δ: 0–4 Hz), (θ: 4–8 Hz), (α: 8–12 Hz), (β: 12–28 Hz), and (γ: 28–100 Hz) (27, 28).

Oxygen was recorded using an OxyLite Pro (Oxford Optronix Ltd.). Oxygen recordings were obtained using an implantable fiber-optic oxygen-sensing device. Light pulses (525 nm) induce fluorescence (measured at 650 nm) at the platinum tip that is quenched by oxygen within a local area (∼0.5–1.0 mm^3^) and uses the fluorescence decay time to derive the partial pressure of oxygen (${\text{P}}_{{\text{O}}_{2}}$) (29). Po_2_ is important because it is directly related to how much free oxygen is present in tissue, thereby representing their oxygenation levels. The technology (Oxylite, Oxford Optronics, UK) does not consume oxygen while measuring absolute ${\text{P}}_{{\text{O}}_{2}}$ values. The optode was chronically implanted under stereotaxic control in the dorsal hippocampus. We allowed five days between implantation and initiation of measurements to ensure that the effects of acute implantation trauma were minimized. ${\text{P}}_{{\text{O}}_{2}}$ measurements at 1 Hz were then be made by connecting the implant to the Oxylite using an extension fiber optic lead. The probe provides accurate and continuous measurements of local ${\text{P}}_{{\text{O}}_{2}}$ levels in brain tissue in awake, freely moving rodents.

Hippocampal “hyperoxia” was defined as oxygen levels exceeding the hyperoxic threshold of 30 mmHg, the upper range of ${\text{P}}_{{\text{O}}_{2}}$ levels previously recorded over several hours in six noninjured rats (16).

### Statistics

EEG power was computed using Spike 2 version 7 from Cambridge Electronic Design Limited. Statistical analysis of EEG power was computed using dependent samples *t* tests with seizure status as the investigated factor. Statistical analysis for oxygen curves was completed using one-way ANOVAs with seizure status as the investigated factor. Statistical analysis for behavioral analysis was completed using three-way ANOVAs with injury (RmTBI; sham), electrographic seizure (seizure; no-seizure) and Bay K8644 (Bay K8644; vehicle) as factors. Tukey honestly significant difference (HSD) post hoc follow-up pairwise comparisons were conducted where applicable. All statistical analyses were conducted using SPSS 20.0 for Mac and considered significant if *P* < 0.05. For Figs. 3, 4, and 5, graphs are displayed as means ± 95 confidence interval (CI), whereas Fig. 6 displays means ± SE. Sample sizes were derived to comply with animal ethics guidelines recommending replacement, reduction, and refinement, in line with our previously published studies demonstrating robust effect size. Data can be found on open source framework at https://www.doi.org/10.17605/OSF.IO/S8FDZ.

## RESULTS

### Posttraumatic Hippocampal Electrographic Seizures and EEG Flattening

**Figure 2.**
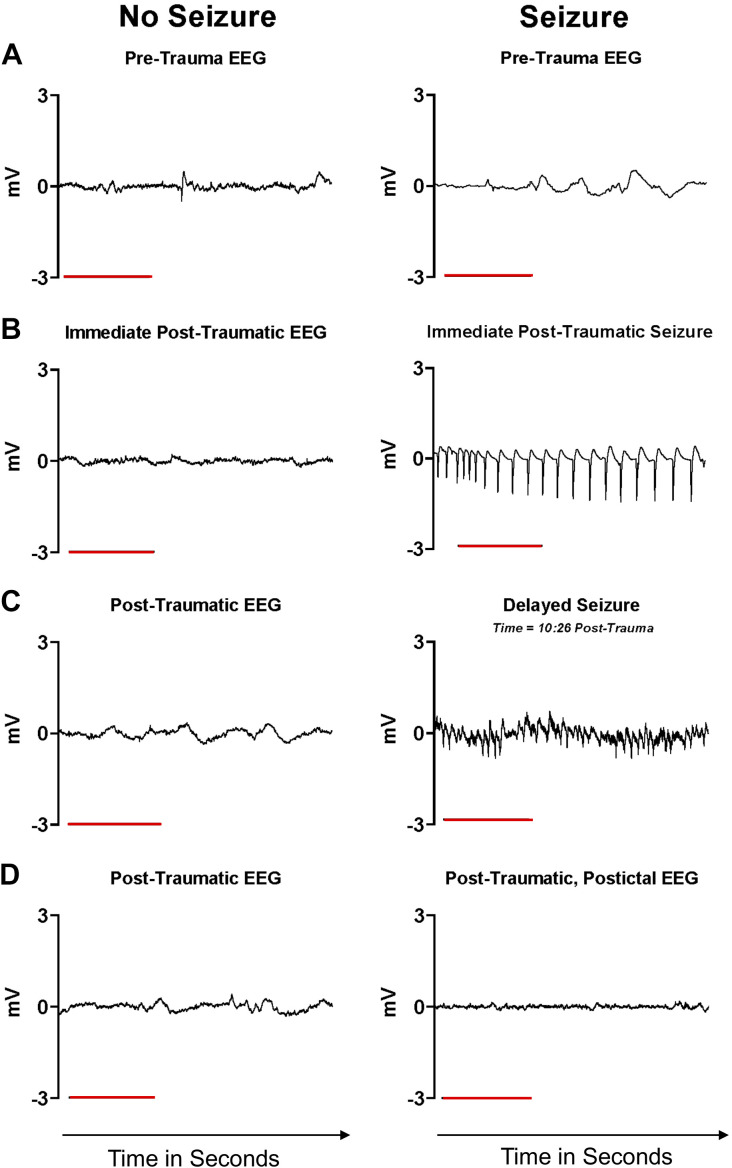
Trauma-induced seizures and subsequent EEG quiescence. *A*: representative EEG tracings from rats before delivery of any traumatic brain impacts. *B*: some rats demonstrate nonbehavioral, electrographic, high amplitude, low frequency seizures within the first 30 s after trauma that last between 15 and 45 s. *C*: in the 24 h following trauma, spontaneous epileptiform activity arose in rats that had immediate posttraumatic seizures, which we have termed delayed seizures. *D*: while all rats display reduced EEG activity posttrauma, rats that manifested immediate posttraumatic seizures also displayed flattening in the posttraumatic postictal period. All horizontal scale bars represent 16 s (located in the *bottom left* portion of the tracings).

To evaluate hippocampal electrical activity in the acute period immediately following RmTBI, we recorded EEG from the ventral hippocampus before, immediately after, and for 30 min following the impacts. After delivery of each impact, a proportion of rats manifested electrographic seizures but, in every case, without behavioral (clinical) seizures (Fig. 2*B*) within the first 30 s posttrauma, that we have termed immediate, posttraumatic seizures. A seizure was defined as an episode of rhythmic spiking activity that was three times the baseline amplitude and a frequency > 5 Hz that lasted at least 10 s. However, the electrographic seizures we observed ranged between 15 and 45 s in duration. As the number of injuries accumulated in the RmTBI protocol, the proportion of rats that displayed these immediate, posttraumatic electrographic seizures increased (25% following the 1st mTBI, 38% following the 2nd mTBI, and 45% following the 3rd mTBI). In the hour following the trauma, ∼1/4 of rats also exhibited interspersed bouts of seizure activity not immediately following the trauma, which we have termed delayed seizures (Fig. 2*C*).

**Figure 3.**
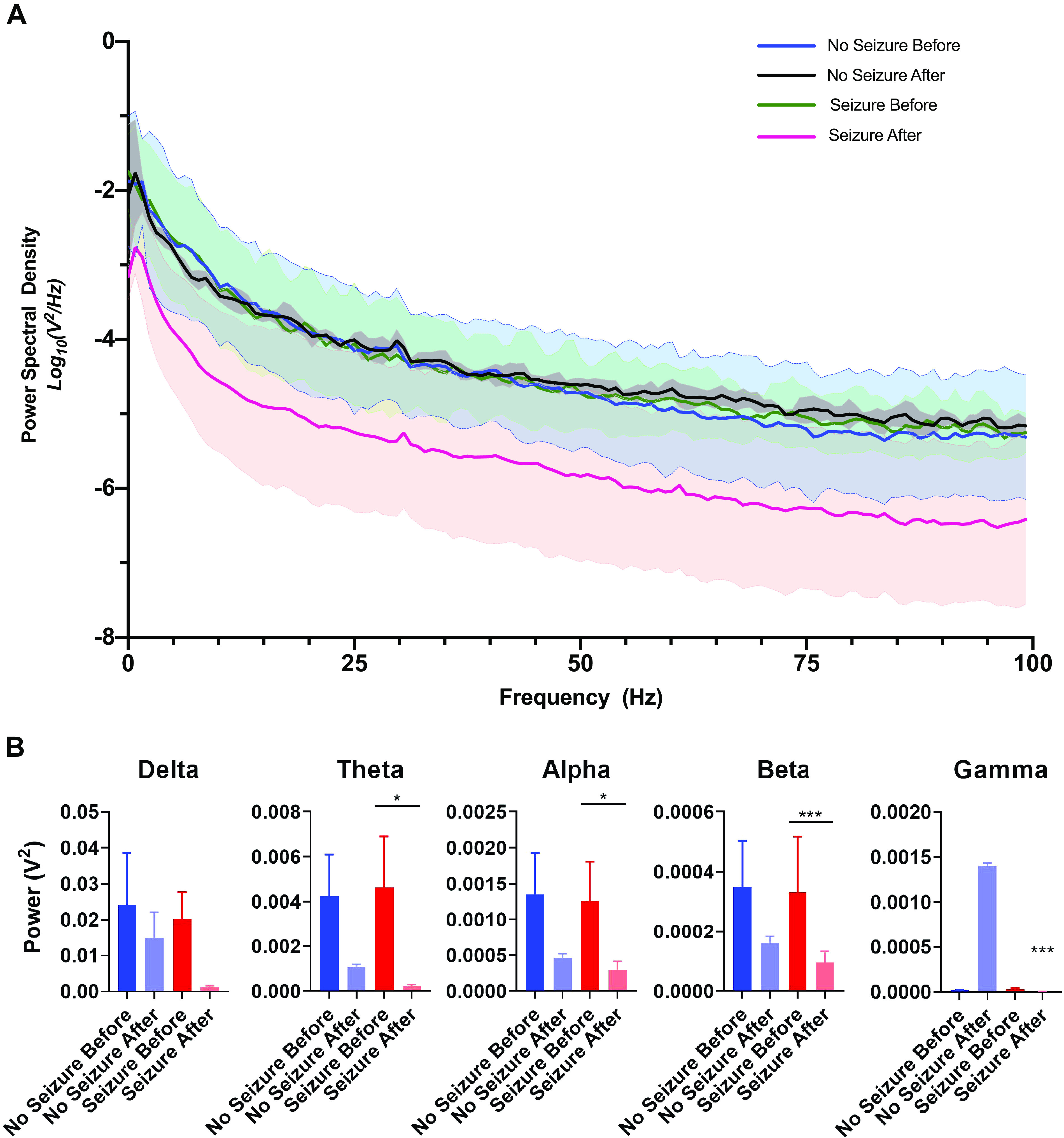
Immediate posttraumatic electrographic seizures result in decreased EEG power. *A*: log transformation of signal power distribution along the range of evaluated frequencies before and after trauma, including 95% confidence intervals. The “no seizure before” curve is the analysis of pretraumatic EEG from rats that did not go on to have an immediate posttraumatic seizure. The “no seizure after” curve is the analysis posttraumatic EEG from those same rats. Similarly, the “seizure before” group is the analysis of pretraumatic EEG from rats that did have an immediate posttraumatic seizure, and the “seizure after” is the posttraumatic EEG analysis of those same rats after they’ve undergone an mTBI and had a seizure. *B*: The power spectrum were separated and quantified in bins corresponding to brain wave frequencies: (δ: 0–4 Hz), (θ: 4–8 Hz), (α: 8–12 Hz), (β: 12–28 Hz), and (γ: 28–100 Hz). Error bars represent means ± standard error. *P < 0.05; ***P < 0.001. No Seizure Before (*n* = 5), No Seizure After (*n* = 5); Seizure Before (*n* = 7), Seizure After (*n* = 7).

Following the trauma and an electrographic seizure, EEG activity recorded from the hippocampus decreased in amplitude (Fig. 2*D*). We conducted a power analysis of the EEG signal before and 3 min after trauma, which showed a dramatic reduction in power across the evaluated frequency spectrum (Fig. 3). The group sizes for this portion of the experiment were: No Seizure Before (*n* = 5), No Seizure After (*n* = 5); Seizure Before (*n* = 7), Seizure After (*n* = 7). Decomposition and quantification of the EEG signal into the functionally distinct frequency domains, (0–4 Hz), (4–8 Hz), (8–12 Hz), (12–30 Hz), and (30–100 Hz), confirmed a significant posttraumatic reduction of band power across 4–100 Hz in rats that exhibited an electrographic seizure after trauma (Fig. 3*B*). Although it appears that rats that did not have a seizure also showed a reduction, although less dramatic, the difference was not significant (Fig. 3*B*).

### Posttraumatic and Postictal Oxygenation

**Figure 4.**
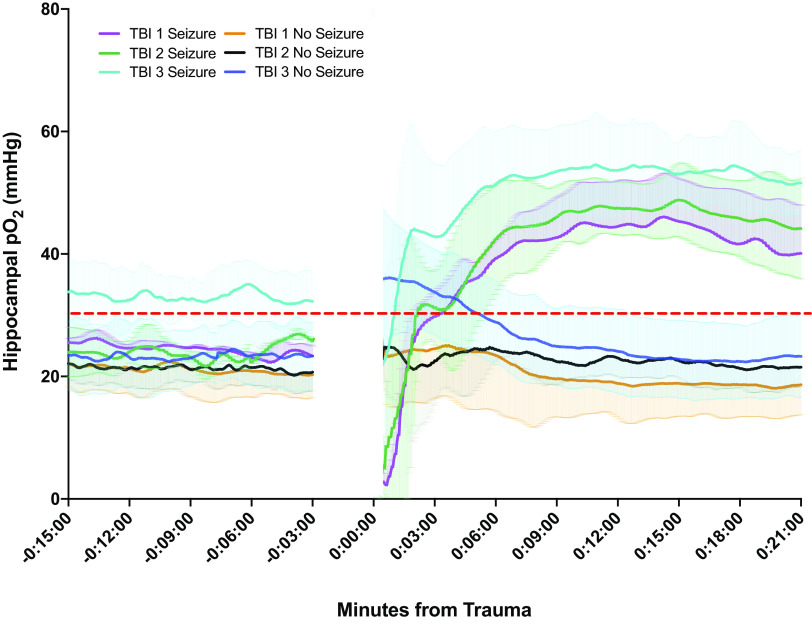
Rats that have posttraumatic seizures demonstrate a different brain oxygen trajectory than rats that undergo mild traumatic brain injury (mTBI) but do not have a seizure. Illustrative representation of hippocampal brain oxygen through each of the mTBIs in the injury protocol. Error bars represent means ± 95% confidence intervals. TBI 1 Seizure (*n* = 5), TBI 1 No Seizure (*n* = 6); TBI 2 Seizure (*n* = 5), TBI 2 No Seizure (*n* = 6); TBI 3 Seizure (*n* = 6), TBI 3 No Seizure (*n* = 5).

Oxygen was recorded from the dorsal hippocampus simultaneously with EEG over the course of the RmTBI protocol. A pretrauma baseline was recorded before delivery of the mTBI and acute posttraumatic oxygen monitoring. After the mTBI, rats that did not have a seizure exhibited a slight, acute elevation in brain oxygen before they returned to normoxic levels below the hyperoxic threshold. Rats that did display an electrographic seizure manifested an acute period of reduced hippocampal oxygenation immediately after delivery of the mTBI that corresponded with the electrographic seizure, followed by a period of hyperoxia (Fig. 4). The difference between hippocampal oxygenation between seizure and no seizures groups were statistically significant after ∼6 min post-mTBI for all mTBIs. The group sizes for this portion of the experiment were: TBI 1 Seizure (*n* = 5), TBI 1 No Seizure (*n* = 6); TBI 2 Seizure (*n* = 5), TBI 2 No Seizure (*n* = 6); TBI 3 Seizure (*n* = 6), TBI 3 No Seizure (*n* = 5).

### Bay K8644 Attenuates Posttraumatic Hyperoxia

**Figure 5.**
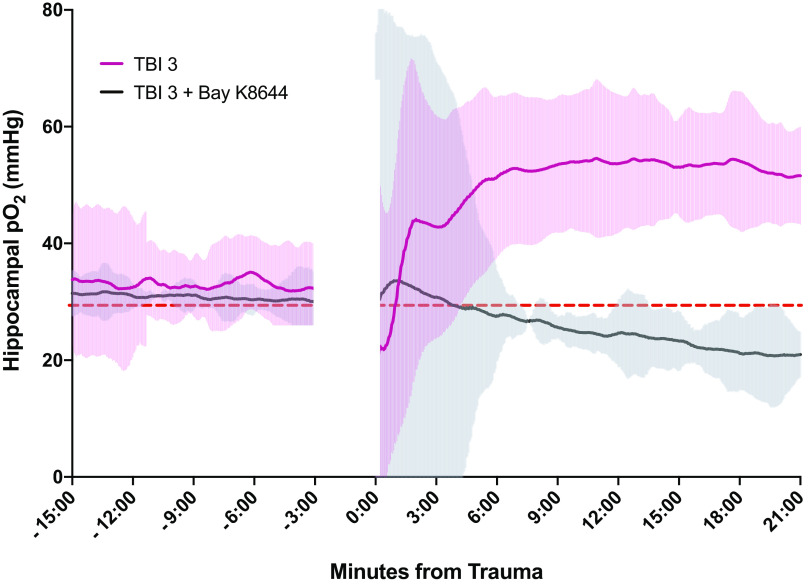
Administration of Bay K8644 before the third injury to rats that have exhibited a history of seizures in the previous two traumatic brain injuries (TBIs). Hippocampal oxygenation through TBI 3 (data from Fig. 4) and TBI 3 with the administration of Bay K8644. Error bars and shaded areas represent ± 95% confidence intervals. The dashed red line at 30 mmHg represents the hyperoxic threshold. TBI 3 (*n* = 4), TBI 3 + Bay K8644 (*n* = 3).

We attempted to ameliorate posttraumatic and postictal hyperoxia by administering the L-type calcium channel agonist, Bay K8644. Rats underwent mTBIs 1 and 2 as per the aforementioned experimental paradigm and were monitored for whether or not they had an electrographic seizure. All rats that had a posttraumatic electrographic seizure to the first impact continued to display posttraumatic seizures to subsequent impacts. Consequently, rats that exhibited an electrographic seizure by mTBI 2 would also have had an electrographic seizure after mTBI 3 and Bay K8644 was administered either immediately before or immediately after the third mTBI. Oxygen traces from rats that had a seizure after mTBIs 1 and 2 followed the same trajectory as previously described in Fig. 4 (with Bay K8644 in Fig. 5). In the group receiving Bay K8644, an initial 6-min period of variable hyperoxia was observed after mTBI, followed by a reduction to slightly below baseline levels and maintenance in the normoxic zone (Fig. 5). The group size reported in Fig. 5 for rats with seizures at all three timepoints with and without Bay K8644 were—TBI 3 no treatment (*n* = 4), TBI 3 + Bay K8644 (*n* = 3).

### Bay K8644 Rescues Posttraumatic Behavioral Dysfunction

**Figure 6.**
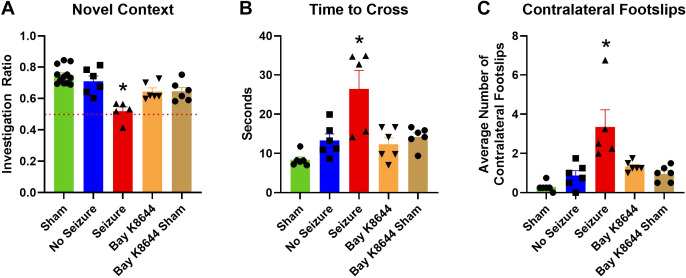
Behavioral deficits between experimental groups. *A*: rats that received repetitive mild traumatic brain injury (RmTBI) and had a seizure (RmTBI + seizure, *n* = 5) performed significantly worse than sham rats (Sham, *n* = 12), rats with RmTBI but no seizure (RmTBI + No seizure, *n* = 6), rats that had a seizure and received Bay K8644 (RmTBI + seizure + Bay K8644, *n* = 6), and sham rats that received Bay K8644 (Bay K8644 + Sham, *n* = 6) in the Novel Context Mismatch (NCM) task. *B*: rats that received RmTBI and had a seizure (RmTBI + seizure, *n* = 5) took significantly longer to cross the beam in the beam-walk task than sham (Sham, *n* = 6), rats with RmTBI but no seizure (RmTBI + No seizure, *n* = 6), rats that had a seizure and received Bay K8644 (RmTBI + seizure + Bay K8644, *n* = 6), and sham rats that received Bay K8644 (Bay K8644 + Sham, *n* = 6). *C*: rats that received RmTBI and had a seizure (RmTBI + seizure, *n* = 5) showed significantly greater contralateral hindleg footslips in the beam-walk task than sham (Sham, *n* = 6), rats with RmTBI but no seizure (RmTBI + No seizure, *n* = 6), rats that had a seizure and received Bay K8644 (RmTBI + seizure + Bay K8644, *n* = 6), and sham rats that received Bay K8644 (Bay K8644 + Sham, *n* = 6). Data are depicted as means with error bars representing means ±standard error. *P < 0.05.

Rats that had RmTBI and subsequent electrographic seizures exhibited significant impairments in all behavior measures evaluated. Performance between sham rats and rats with RmTBI but without seizures, did not significantly differ in the NCM, time to cross the beam, nor in footslips in the beam-walk task (Fig. 6). Rats that were administered Bay K8644 after RmTBI and seizures (RmTBI + seizure + Bay K8644) were statistically indistinguishable from the sham and the RmTBI without seizure groups. There was a drug effect in the NCM, where sham rats that were administered Bay K8644 (Bay K8644 + sham + no seizure) differed from sham rats but not from the RmTBI + seizure group. Sham rats who received Bay K8644 (Bay K8644 + sham + no seizure) did not differ significantly from rats who received Bay K8644 (RmTBI + seizure + Bay K8644). In summary, significant behavioral impairments were identifed in rats that manifested posttraumatic electrographic seizures (RmTBI + seizure) as exhibited by poorer short-term working memory in the NCM task and poorer motor coordination in the beam-walk task (took longer to cross the beam and made more foot placement errors). The group sizes for this portion of the experimental analyses can be found in Fig. 6 legend.

## DISCUSSION

### Electrographic Findings in the Immediate Phase after mTBI

Using in vivo recording techniques in awake, freely moving rats, and following the delivery of a lateral impact to induce RmTBIs, we detected localized electrographic seizures, followed by a period of EEG dampening in the hippocampus. Behavioral (clinical) seizures that are discernable by gross observation alone were not witnessed. Consequently, it may be the case that posttraumatic electrographic, but not behavioral seizures, are occurring, undetected, in a certain proportion of the clinical population that experiences mTBI. It is possible that this series of events is triggered by the synchronized stretching of axons as the brain accelerates and decelerates during the injury process (30). Axonal stretching and membrane disruption produces coordinated, nonspecific depolarization and increases in membrane conductance, which may be responsible for the changes in excitability and the resultant electrographic seizure activity (31). The immediate postictal period of EEG dampening is a common observation indicative of a preceding electrographic seizure and is likely the continued manifestation of seizure terminating mechanisms (32).

Although this is the first study on the immediate electrographic manifestation of mild TBIs, subacute studies on severe TBI have observed changes in excitability and circuit function (33). Consequently, postinjury changes in brain activation have been postulated to manifest phenotypically as deleterious changes to cognitive and affective well-being (34). It is therefore possible that the electrographic seizure activity we observed following mTBI may be an indication of the same types of changes to neuronal excitability as previously identified in more severe TBIs. Consequently, this phenomenon may be a key tenet of the pathophysiological underpinnings of the behavioral deficits manifested by people who have sustained an mTBI, that was accompanied electrographic seizures without behavioral (clinical) seizures.

Power analysis of pre- and posttraumatic EEG revealed a profound dampening of the EEG signal in rats that had an electrographic seizure after mTBI. The most significant dampening of the EEG signal occurred in the 12–100 Hz frequency domains, with a statistically significant difference between posttrauma seizure and posttrauma no seizure groups. The postictal dampening of these brain frequencies is likely the fingerprint that posttraumatic electrographic seizures temporarily leave on brain activity. In humans, β and γ frequencies are thought to be involved in cognitive functioning, learning, memory, and information processing (34, 35). That is, disorders that result in the chronic manifestation of concentration, memory, executive function, and mood dysfunction display suppression of the EEG signal in the same domains as those observed after posttraumatic seizures. Coincidentally, posttraumatic electrographic seizures occur after an event that is also associated with many of the same behavioral impairments. This commonality may hint at a shared mechanism behind the chronic symptomology of some neurological disorders and the acute/subacute deficits that follow concussions. In the case of concussions, it is plausible that the trigger for these events is posttraumatic electrographic seizures.

Just as there is individual heterogeneity in mTBI manifestations, where not every mTBI produces the same severity of symptomology across populations (36, 37), not all mTBIs produced posttraumatic electrographic seizures in our study. Although the proportion of experimental rodents that exhibited posttraumatic seizures increased as the number of successive injuries accumulated, not all rodents were susceptible. This is consistent with reports, both in preclinical models and in human populations, of postconcussive vulnerability to further injury, particularly in cases where a second concussive injury is sustained within days of the first, leading to more severe deficits (33). It is possible that individuals with the worst outcomes after mTBI are, in fact, the individuals that had posttraumatic electrographic seizures. Thus, the reason why individuals are more vulnerable to worse symptoms after RmTBIs may be because successive injuries sensitizes the brain and places one at greater risk for the generation of posttraumatic electrographic seizures, than single isolated mTBIs. The initial manifestation of an electrographic seizure resulted in the priming of neuronal networks for synchronicity—that is, the generation of a “focus” for the propagation of further electrographic seizure activity (38). Consequently, after an initial electrographic seizure, the threshold required to produce another one is lowered, and it becomes easier to induce subsequent seizures. The epidemiological findings that a single concussion increases risk for future concussions may support our findings that electrographic seizures are an important aspect of mTBI pathophysiology.

### Brain Oxygenation after mTBI

It has been contended that concussion and mTBI are metabolic rather than structural injuries, wherein a metabolic mismatch between energy demand and energy supply results in cellular vulnerability that is particularly susceptible to further injury (39). This theory is in congruence with our electrographic findings: a seizure involves a period of heightened metabolic demand and this period of metabolic mismatch has been related to behavioral impairments consistent with a failure to meet oxygen demands of the brain. Investigations on oxygen and blood flow in the postictal period of regions of the brain that have exhibited single brief self-terminating seizures have shown a period of hypoxia, that, when reversed, prevents the behavioral impairments that follow seizures (16). Whereas prolonged or rapidly repeating seizures (status epilepticus) that results from damage are associated with hyperoxia (14).

To our knowledge, this is the first study to report on the oxygen status of nonneocortical brain structures immediately after a mild TBI. We observed an increase in brain oxygen in the hippocampus immediately after the first injury and posttraumatic electrographic seizures, culminating in a period of hyperoxia wherein oxygen levels were 1.5-fold above baseline levels; gradually resolving in the following 24 h. Following the second injury and posttraumatic seizures, the hyperoxia lasted at least 48 h. Interestingly, rats that received an mTBI but did not demonstrate posttraumatic electrographic seizures did not exhibit this phenomenon of posttraumatic, postictal hyperoxia, leading us to conclude that the changes in oxygenation were a result of the posttraumatic seizures, and not the mTBI itself.

This change in oxygenation does not follow the typical trajectory of brain tissue that has undergone a single self-terminating seizure (16), and is more like brain damaged induced status epilepticus that leads to hyperoxia. Moreover, it seems that post-mTBI oxygenation in the hippocampus follows the opposite trend as that identified in the neocortex following severe TBI. That is, while after severe TBI, the neocortex exhibits hypoxia that gradually resolves over the first day, but following mTBI the hippocampus exhibits hyperoxia that resolves over the same period (40). If mTBIs cause wide ranging hyperoxia, it is possible that mTBIs are fundamentally different from severe TBIs in their pathophysiology and therefore have different vascular and oxygenation implications. The diffuse and nonmacroscopic damage sustained in mTBIs may result in the activation of different mechanisms and pathways than the much more destructive pathology seen in severe TBIs. Similarly, different seizures may result in different physiological outcomes. Prolonged seizures (status epilepticus) induced by either long-term electrical stimulation or kainic acid result in hippocampal hyperoxia both during the status event and for weeks after (17). While it may be that brain damage from a number of different causes result in a signal that drives vasodilation and in turn leads to hyperperfusion and hyperoxia, it may also be the case that damaging seizures have the same result of vasodilation and hyperoxia.

### The Effect of Changes in Oxygenation on Immediate Behavioral Outcomes

Rats that demonstrated posttraumatic electrographic seizures and increases in posttraumatic brain oxygenation demonstrated marked deficits in short-term working memory and motor function compared with sham rats and rats that received a mTBI but did not manifest posttraumatic seizures or hyperoxia. In fact, rats that received the injury but did not have seizures or have substantial changes in brain oxygenation performed similarly to rats that did not receive any injury at all. It is therefore possible that the true culprit, at least with respect to the behavioral measures tested in this study, is not the physical trauma itself, but the ensuing seizure and vascular events that follow. This is consistent with recent assertions in the field claiming that concussions and other mTBIs are metabolic and not structural injuries (7).

If it is these deviations in blood supply and oxygenation in the brain that cause posttraumatic behavioral deficits, blocking this abnormal pathophysiological response should attenuate the deficits. Indeed, treatment with a vasoconstrictive agent, Bay K8644, to restore vascular tone, reduced the extent of hyperoxia after injury and rescued the deficits demonstrated by rats after RmTBI, although we acknowledge that L-type calcium channel agonists could have a variety of effects, such as the facilitation of neurotransmitter release, not related to vasoconstriction (41). Bay K8644 is likely acting centrally rather than peripherally as this L-type calcium channel agonist, at the dosage given, would have raised arterial blood pressure and likely increased rather than decreased hippocampal oxygen levels. Bay K8644 has been shown to have proconvulsant effects when administered as an adjunct to other convulsant chemical agents and at higher dosages than used in this study (42). Our data provides evidence that restoring brain oxygen to normoxic levels may be a viable therapeutic option for posttraumatic deficits. In broader terms, it seems that dysregulation of physiologic parameters such as oxygenation may be a key aspect of the behavioral deficits commonly observed following mTBI. Consequently, it may be useful to investigate ways by which restoring the autoregulatory capacity of the brain as a method to combat the severity and length of posttraumatic deficits.

The changes in oxygenation may also present a rationale for why there is heterogeneity in mTBI patient symptomologies. It is already established that different impact locations result in the manifestation of different deficits (21). Different regions in the brain may experience perturbations in oxygenation depending on the trauma impact location and other injury dynamics, resulting in different deficits based on which brain structures are affected. Thus, restoration of normal autoregulatory function in the brain is paramount to overcoming posttraumatic deficits. Moreover, it is also possible that posttraumatic, postictal hyperoxia is not the only contributor to posttraumatic deficits. Posttraumatic electrographic seizures may result in a plethora of downstream cellular events beyond changes in vascular tone that may be partly responsible for neurological dysfunction and behavioral deficits.

### Conclusions

This study reported the presence of an immediate, electrographic posttraumatic seizure, and subsequent hyperoxic episode, that when reversed, rescued the behavioral deficits identified after RmTBI. We propose that the posttraumatic seizure is an important part of what differentiates a symptomatic mTBI, or concussion, from one without persistent symptomology, and that hemodynamic perturbations resulting in changes to oxygenation of the brain are key etiological factors for many of the deficits observed after concussion. Future studies should evaluate blood flow and oxygenation changes at the level of the whole brain to ascertain the extent of homeostatic dysregulation after concussion and attempt to correlate changes in oxygenation of certain brain structures to corresponding deficits. Given that the optode and electrode were implanted into the hemisphere contralateral to injury it is possible that our findings are an underestimate of the actual changes in electrographic activity and oxygenation in the impacted hemisphere. In addition, this study is limited because it only used male rats to characterize the presented phenomena. As estrogen acts as both a genomic and nongenomic neuroprotectant (43), it is possible that increased estrogen in females would protect them from electrographic seizure. Conversely, evidence does suggest that testosterone possesses anti-inflammatory properties and reduces susceptibility to cortical spreading depression (44, 45), suggesting that the electrographic effects may have been larger in females. Future studies should look to characterize this in both sexes as it may contribute to the sex-dependent heterogeneity in postconcussive symptomology. In addition, the study would have benefitted from immunohistochemical examination of the tissue surrounding the optode and electrode implantation, to ensure that the RmTBIs did not cause additional damage or movement. Nevertheless, determining how to reverse these oxygenation changes may be an important tool in relieving some of the symptoms of mTBI.

## DATA AVAILABILITY

Data can be found on open source framework at https://www.doi.org/10.17605/OSF.IO/S8FDZ.

## GRANTS

The authors thank the Alberta Children’s Hospital Research Institute, Canadian Institute of Health Research Grant PJT-153051 (to R.M.), PJT-152956 (to G.C.T.), and the Natural Sciences and Engineering Research Council Grant 1304881 (to R.M.) for their financial contributions.

## DISCLOSURES

No conflicts of interest, financial or otherwise, are declared by the authors.

## AUTHOR CONTRIBUTIONS

H.M., G.C.T., and R.M. conceived and designed research; H.M. and M.D.W. performed experiments; H.M., M.D.W., and R.M. analyzed data; H.M., G.C.T., and R.M. interpreted results of experiments; H.M. and R.M. prepared figures; H.M. and M.D.W. drafted manuscript; H.M., G.C.T., and R.M. edited and revised manuscript; H.M., M.D.W., G.C.T., and R.M. approved final version of manuscript.
